# Brain tissue oxygen partial pressure monitoring and prognosis of patients with traumatic brain injury: a meta-analysis

**DOI:** 10.1007/s10143-024-02439-4

**Published:** 2024-05-17

**Authors:** Yuqi Shen, Dan Wen, Zhenghua Liang, Li Wan, Qingli Jiang, Haiyan He, Mei He

**Affiliations:** 1 Intensive Care Unit, School of Medicine, Mianyang Central Hospital, University of Electronic Science and Technology of China, Mianyang, Sichuan Province China; 2 Department of Nursing, School of Medicine, Mei He: RN, BSN, Mianyang Central Hospital, University of Electronic Science and Technology of China, No.12 Changjia Alley, Jingzhong Street, Fucheng District, Mianyang, 621000 Sichuan Province China

**Keywords:** Traumatic brain injury, Brain tissue oxygen monitoring, Cerebral perfusion pressure, Intracranial pressure, Meta-analysis

## Abstract

To assess whether monitoring brain tissue oxygen partial pressure (PbtO_2_) or employing intracranial pressure (ICP)/cerebral perfusion pressure (CCP)-guided management improves patient outcomes, including mortality, hospital length of stay (LOS), mean daily ICP and mean daily CCP during the intensive care unit(ICU)stay. We searched the Web of Science, EMBASE, PubMed, Cochrane Library, and MEDLINE databases until December 12, 2023. Prospective randomized controlled and cohort studies were included. A meta-analysis was performed for the primary outcome measure, mortality, following the Preferred Reporting Items for Systematic Reviews and Meta-Analyses guidelines. Eleven studies with a total of 37,492 patients were included. The mortality in the group with PbtO_2_ was 29.0% (odds ratio: 0.73;95% confidence interval [CI]:0.56–0.96; P = 0.03; I = 55%), demonstrating a significant benefit. The overall hospital LOS was longer in the PbtO_2_ group than that in the ICP/CPP group (mean difference:2.03; 95% CI:1.03–3.02; P<0.0001; I = 39%). The mean daily ICP in the PbtO_2_ monitoring group was lower than that in the ICP/CPP group (mean difference:-1.93; 95% CI: -3.61 to -0.24; P = 0.03; I = 41%). Moreover, PbtO_2_ monitoring did not improve the mean daily CPP (mean difference:2.43; 95%CI: -1.39 to 6.25;P = 0.21; I = 56%).Compared with ICP/CPP monitoring, PbtO_2_ monitoring reduced the mortality and the mean daily ICP in patients with severe traumatic brain injury; however, no significant effect was noted on the mean daily CPP. In contrast, ICP/CPP monitoring alone was associated with a short hospital stay.

## Introduction

Traumatic brain injury (TBI) refers to the destruction of the anatomy and physiology of the brain due to external forces. The estimated annual incidence of TBI is 27–69 million cases. Additionally, TBI is one of the leading causes of mortality and morbidity globally, contributing to a substantial economic burden on families and society [1, 2]. Secondary brain injury occurs after TBI due to high oxygen consumption and insufficient cerebral blood flow, which results in cerebral hypoxia. Currently, the treatment of TBI mainly focuses on the prevention of secondary brain injury with the routine monitoring of intracranial pressure (ICP) and maintaining adequate cerebral perfusion pressure (CPP) [3, 4] for the potential goal of maintaining a continuous supply of energy substrates and oxygen to improve neurological outcomes [5]. Currently, it is believed that ICP monitoring-guided treatment can improve the prognosis of patients with severe TBI. Moreover, the guidelines recommend monitoring ICP to calculate and maintain CPP to prevent cerebral ischemia and cerebral infarction [6–8]. However, studies have demonstrated that traditional ICP/ CPP monitoring cannot accurately detect most episodes of hypoxia [9]. Some patients with normal ICP and CPP may still develop cerebral ischemia and hypoxia [10].

Partial pressure of brain tissue oxygen (PbtO_2_) is a monitoring system for detecting the oxygen tension in the brain. Additionally, PbtO_2_ is an indicator that reflects the oxygenation status of the brain by directly measuring the partial pressure of oxygen in the brain using a probe placed within it. The normal range of PbtO_2_ is believed to be 16-40mmHg. Furthermore, PbtO_2_ of 10–15mmHg indicates mild cerebral hypoxia, and PbtO_2_ < 10mmHg indicates severe hypoxia. The purpose of PbtO_2_ is to identify episodes of decreased cerebral perfusion with or without associated raised ICP [11] because the partial pressure of oxygen in the brain can change in the early stages of injury [12]. Changes may also occur before or independently of increases in ICP [13]. PbtO_2_ monitoring has been used to assess, judge and regulate brain hypoxia to guide treatment and improve neurological outcomes [14]. PbtO_2_ can sensitively reflect the blood and oxygen supply to the brain, which is conducive to early detection of cerebral ischemia and hypoxia, and is an independent and sensitive predictor of cerebral ischemia and hypoxia [6]. Additionally, PbtO_2_ monitoring, ICP < 20mmHg, and CPP > 60mmHg have been reported to alleviate cerebral hypoxia through automatic regulation of the brain simultaneously [8].

However, the results of observational and cohort studies and randomized controlled trials remain controversial. Therefore, this meta-analysis was designed to summarize the effectiveness of this approach. The clinical efficacy and safety of PbtO_2_ in patients with TBI were evaluated by comparing the effects of PbtO_2_ (or combined) ICP/CPP-guided treatment and ICP/CPP-guided treatment alone on mortality, length of stay, ICP, and CPP during monitoring.

## Materials and methods

The Preferred Reporting Items for Systematic Reviews and Meta-Analyses (PRISMA) guidelines [15] were followed in the review process and analyses. The study protocol was registered in PROSPERO (CRD42023494630).

### Search strategy

The Web of Science, EMBASE, PubMed, Cochrane Library, MEDLINE databases were searched from inception to December 12, 2023. The search was performed using the following combination of keywords: brain injuries, traumatic or traumatic brain injury or TBI, and brain tissue oxygen or PbtO_2_ and monitoring. Search results were restricted to studies with adult participants published in English.

### Inclusion and exclusion criteria

We used the following inclusion criteria: type of study (randomized or quasi-randomized controlled trials, cohort studies, or case-control studies); type of participants (studies with patients diagnosed with TBI); type of intervention (comparison of PbtO_2_ monitoring alone or combined PbtO_2_ and ICP/CPP monitoring with ICP/CPP monitoring alone); and type of outcome (primary outcome: mortality; secondary outcomes: length of stay in hospital, mean daily ICP, and mean daily CPP). Studies were excluded if they were published in languages other than English or as conference abstracts, case reports, or letters. Studies that included children; studies without sufficient data; and studies that reported data incorrectly (not appropriate for synthesis) were also excluded.

### Study selection and data extraction

We removed duplicate articles from the search results using Endnote (version 9.3). The literature was then screened by two researchers (Yuqi Shen and Dan Wen) according to the inclusion and exclusion criteria outlined above. Disagreements were discussed with a third researcher (Zhenghua Liang) and a consensus was achieved. The extracted data included the first author, publication date, patient care settings, total sample size, intervention measures, and outcome indicators.

### Quality evaluation

We assessed the quality of observational cohort studies using the Newcastle–Ottawa Scale (NOS). A score of 0–4 indicated low-quality literature (grade C), while 5–6 indicated medium-quality literature (grade B), and 7–9 indicated high-quality literature (grade A). The quality of randomized controlled trials was assessed using the Cochrane Risk of Bias Tool. A study was awarded grade A if the standards were completely met, grade B if the standards were partially satisfied, and grade C if none of the standards were met.

### Statistical analysis

Statistical analysis was performed using Review Manager 5.4 (RevMan V5.4.1) provided by the Cochrane Collaboration (Oxford, UK). Odds ratios (ORs) with 95% confidence intervals (CIs) were determined to assess pooled effects. Heterogeneity in the included studies was assessed using Q and *I*^2^ tests. A random-effects model was adopted to analyze variations across the included studies. The stability of the results was assessed using sensitivity analysis by eliminating one study at a time.

## Results

### Study selection

**Fig. 1:**
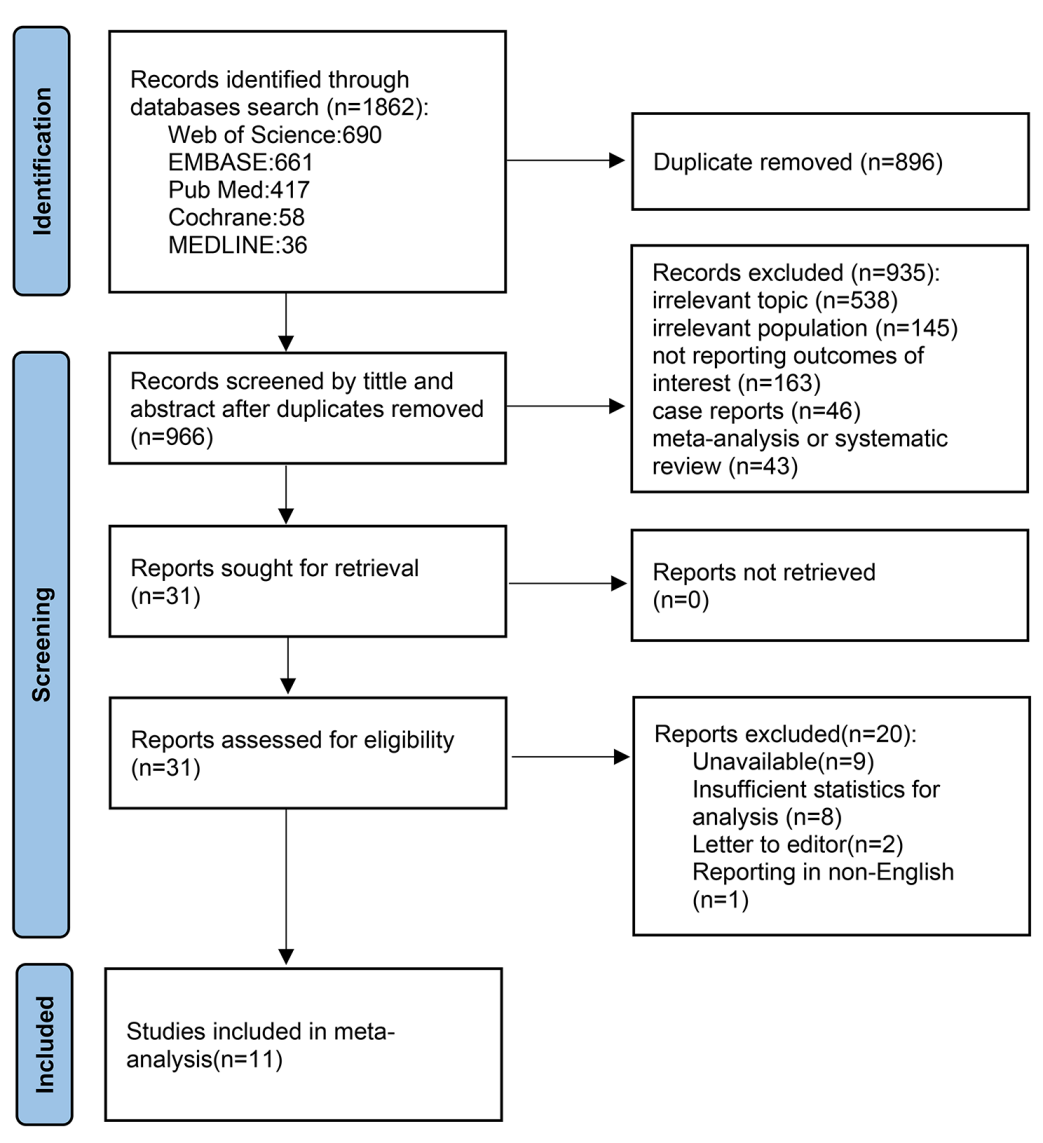
PRISMA flowchart

The PRISMA flowchart (Fig. 1) displays that 1862 articles were originally retrieved; 996 were retained after removing duplicates. During the first screening of titles and abstracts, 935 articles were excluded for the following reasons: irrelevant topic (538 articles), irrelevant population (145 articles), not reporting outcomes of interest (163 articles), case report (46 articles), or meta-analysis or systematic review (43 articles). Eleven articles that met all conditions were identified using full-text screening and included in the final analysis [13, 16–25].

### Study characteristics and quality

The main features extracted from the studies that were included in our analysis are listed in Table 1. We included a total of 37,492 patients, with the sample size ranging from 50 to 35,501 participants per study. On quality assessment, six studies were awarded grade A [13, 18, 20–23], and four were awarded grade B [16, 17, 19, 24, 25]. Details of the assessment of each study are provided in Tables 2 and 3.

**Table 1:** Characteristics and quality of included studies

NO	Study and Year	Country	Design	Clinical setting	*N*	Interventions	Outcome	Measurements testing	Grade
1	Barrit, et al.,2022	Belgium	cohort	ICU	70	C: ICP-only I: ICP + PbtO2-monitored	Mortality; Overall LOS, days;	Not mentioned	A
2	Green, et al.,2012	US	cohort	Level 1 trauma center	74	C: ICP-only I: PbtO2-monitored	Mortality; Overall LOS, days;	Not mentioned	A
3	Hoffman, et al.,2020	US	cohort	The National Trauma Data Bank (NTDB)	465	C: ICP-only I: PbtO2-monitored	Mortality	Not mentioned	A
4	Komisarow, et al.,2021	US	cohort	United States trauma centers	35,501	C: ICP-only I: PbtO2-monitored	Mortality; Overall LOS, days;	Not mentioned	A
5	Lin, et al.,2015	Tai Wan	RCT	6 collaborative hospitals in northern	50	C: ICP + CCP I: ICP + PbtO2-monitored	Mortality; mean daily ICP and CCP (mmHg) during the ICU stay;	Hospitals were offered training courses in the standard protocol.	B
6	Martini, et al.,2009	US	cohort	Harborview Medical Center	629	C: ICP-only I: ICP + PbtO2-monitored	Mortality; Overall LOS, days; mean daily ICP and CCP (mmHg) during the ICU stay	Not mentioned	A
7	McCarthy, et al.,2009	US	cohort	Level I trauma center (Miami Valley Hospital, Dayton, OH)	145	C: ICP + CCP I: ICP + PbtO2-monitored	Mortality; Overall LOS, days;	Not mentioned	A
8	Meixensberger, et al.,2003	Germany	cohort	in the NICU at the Würzburg University Hospital	93	C: ICP + CCP I: ICP + PbtO2-monitored	mean daily ICP and CCP (mmHg) during the ICU stay	Not mentioned	B
9	Spiotta, et al.,2010	US	cohort	a NICU at a university-based Level I trauma center and tertiary care hospital	123	C: ICP + CCP I: PbtO2-monitored	Mortality; Overall LOS, days; mean daily ICP and CCP (mmHg) during the ICU stay	Not mentioned	B
10	Stiefel, et al.,2005	US	cohort	NICU	53	C: ICP + CCP I: PbtO2-monitored	Mortality; mean daily ICP and CCP (mmHg) during the ICU stay	Not mentioned	B
11	Payen, et al.,2023	France	RCT	ICU of 25 French tertiary referral centres	289	C: ICP I: ICP + PbtO2-monitored	Mortality	specific training was provided to local clinicians	B

**Table 2:** Quality and risk of bias assessment using the Newcastle-Ottawa Scale (NOS) for observational studies

Study ID	Selection				Comparability	Outcome			Total (9*)
	Representativeness of the exposed cohort (*)	Selection of non-exposed cohort (*)	Ascertainment of exposure (*)	Demonstration that outcome of interest was not present at start of study (*)	Comparability of cohorts (**)	Assessment of outcome (*)	Length of follow up(*)	Adequacy of follow up (*)	
Barrit, et al.2022	*	*	*	*	*	*	*	*	8
Green, et al.2012		*	*	*	*	*	*	*	7
Hoffman, et al.2020	*	*	*	*	*	*	*	*	8
Komisarow, et al.2021	*	*	*	*	*	*	*	*	8
Martini, et al.2009	*	*	*	*	*	*		*	8
McCarthy, et al.2009	*	*	*	*	*	*	*	*	8
Meixensberger, et al.2003			*	*	*	*	*	*	6
Spiotta, et al.2010	*		*	*	*	*		*	5
Stiefel, et al.2005	*		*	*	*	*		*	6

**Table 3:** Quality of evidence (GRADE) and of the risk of bias assessment by using the Cochrane ROB tool 2 for randomized clinical trials

Study ID	Randomization process	Deviation from the intended interventions	Missing outcomes data	Measurement of the outcome	Selection of the reported results	Overall ROB	Quality of level GRADE
Lin, et al.,2015	Some concern	Low	Some concern	Low	Low	Some concern	Moderate
Payen, et al.,2023	Low	Low	Some concern	Low	Low	Some concern	Moderate

### Primary outcome: mortality

**Fig. 2:**
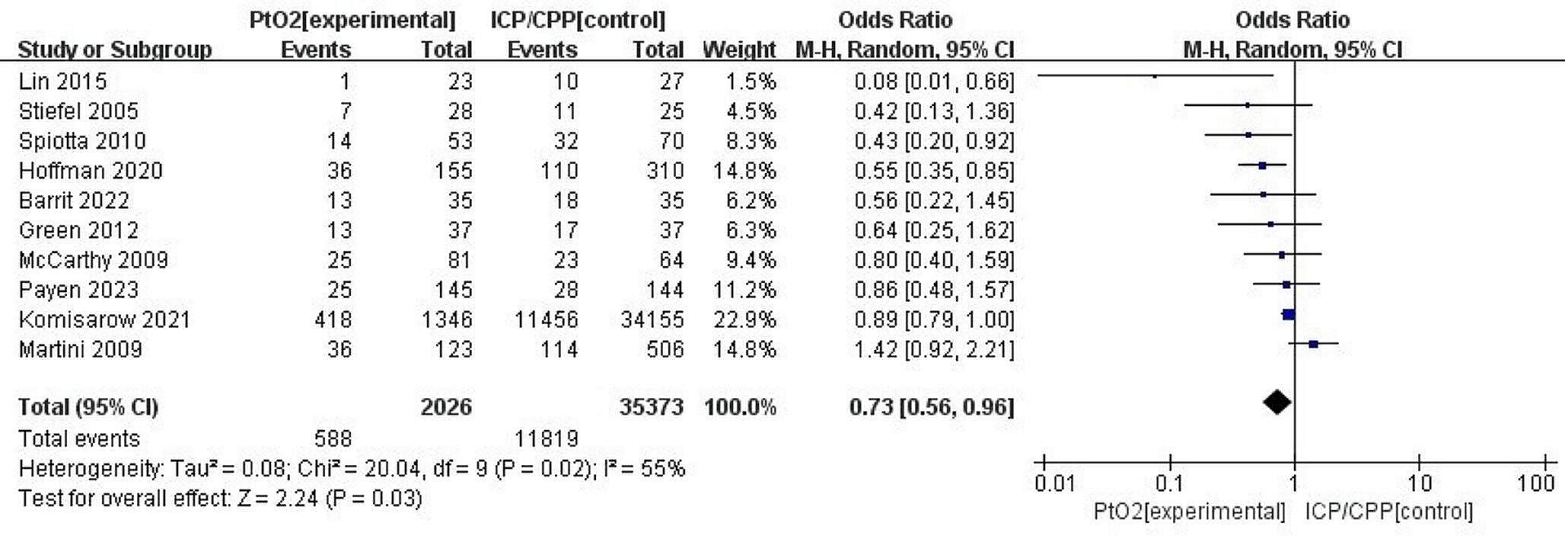
Forest plot the effects of PbtO2 monitoring towards mortality

The mortality rate was 29.0% in 2026 patients with TBI in the PbtO_2_ monitoring group. The OR for mortality in the PbtO_2_ monitoring group, compared to that of ICP/CPP, was 0.73 (95% CI: 0.56–0.96; *P* = 0.03; *I*^2^ = 55%), thus demonstrating a significant benefit (Fig. 2).

### Secondary outcomes

**Fig. 3:**
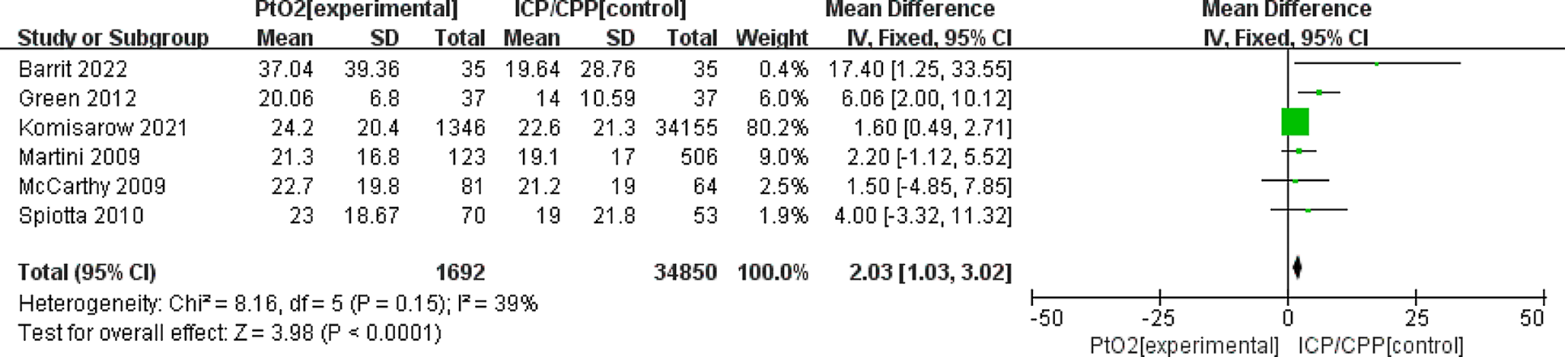
Forest plot the effects of PbtO2 monitoring towards overall LOS

The mean difference(MD)in the length of hospital stay in patients in the PbtO_2_ monitoring group (reported in six studies) was 2.03 (95% CI: 1.03–3.02; *P<*0.0001; *I*^2^ = 39%);therefore the overall length of stay in the hospital in the ICP/CPP monitoring group was shorter than that in the PbtO_2_ monitoring group (Fig. 3).

**Fig. 4:**
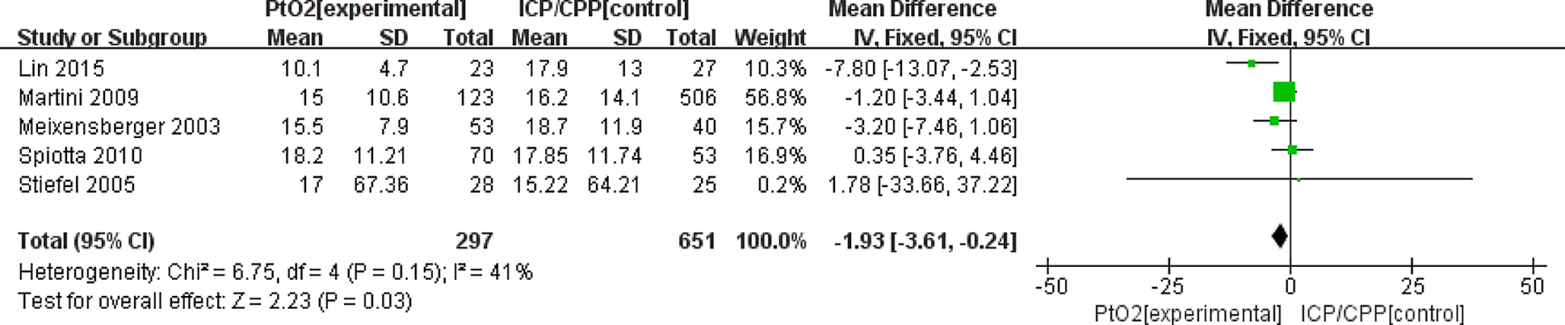
Forest plot the effects of PbtO2 monitoring towards mean daily ICP during the ICU stay

The MD in the mean daily ICP between PbtO_2_ monitoring group and ICP/CPP monitoring group was − 1.93 (95%CI: -3.61 to -0.24; *P* = 0.03; *I*^2^ = 41%)(based on five studies). The mean daily ICP in the PbtO_2_ monitoring group was lower than that in the ICP/CPP monitoring group (Fig. 4).

**Fig. 5:**
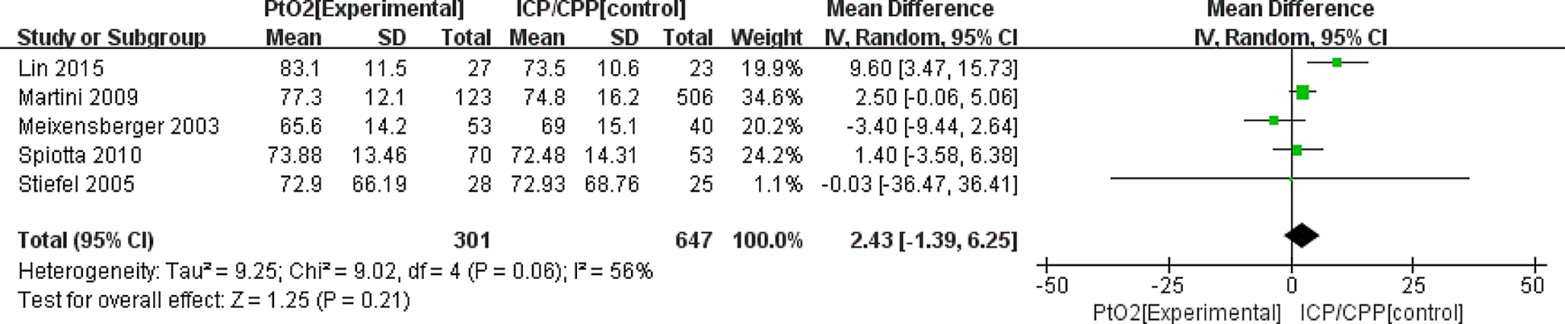
Forest plot the effects of PbtO2 monitoring towards mean daily CPP during the ICU stay

The MD in the mean daily CPP between the PbtO_2_ monitoring group and ICP/CPP monitoring group was 2.43 (95%CI: -1.39 to 6.25; *P* = 0.21; *I*^2^ = 56%), thus demonstrating a non-significant benefit(based on five studies). PbtO_2_ monitoring did not improve the mean daily CPP (Fig. 5).

## Discussion

We pooled the results of nine retrospective cohort studies and two randomized controlled trials involving 37,492 patients with TBI, including 2,096 in the PbtO_2_ monitoring group and 35,396 in the ICP/CPP monitoring group. Our primary meta-analysis investigated the impact of brain tissue oxygen monitoring in patients with TBI compared to standard ICP/CPP monitoring in terms of mortality; hospital length of stay; and mean daily ICP and CPP.

Our results demonstrated that the role of PbtO_2_ monitoring in reducing mortality remains clear. Four studies included in this meta-analysis reported that PbtO_2_ monitoring does not decrease a significant mortality rate. In contrast, seven studies identified a reduction in the risk of mortality. The rate of mortality in the PbtO_2_ monitoring group was 29.0% across all studies included in our analysis, which is different from the previously reported rate based on a meta-analysis by Xie [4]. Studies have demonstrated that brain hypoxia is associated with poor prognosis after TBI and optimizing PbtO_2_ can improve recovery and survival rates [26]. Four observational studies [19, 20, 22, 23] and two randomized controlled trials [16, 25] included in this meta-analysis demonstrated that when PbtO_2_ was incorporated into clinical management decisions, neural function exhibits statistically significant benefits. Additionally, five studies demonstrated that PbtO_2_ monitoring increased mortality in patients with TBI. Although no single treatment has been identified to significantly decrease the mortality of these patients in a relatively short period, improving patient outcomes through corresponding interventions is possible.

PbtO_2_ monitoring group was associated with prolonged the hospital length of stay; however, it is worth noting that three reports included in this analysis revealed a significant difference in hospital length of stay, while three other studies indicated no such difference between patient groups. However, our meta-analysis results revealed that PbtO_2_ monitoring was associated with extended hospital length of stay. Although the severity of TBI at admission was comparable between patient groups, the difference may be attributed to patients in the PtbO_2_ monitoring group receiving more intensive life-support treatments, potentially leading to prolonged survival [18]. The relationship between PbtO_2_ monitoring and extended hospital length of stay in patients with TBI, therefore, needs further evaluation.

Only one study reported a significant relationship between the mean daily ICP and CPP with PbtO_2_ monitoring, while four studies reported no significant association. Our meta-analysis suggests that ICP can be reduced further with PbtO2 monitoring. However, mean daily CPP is demonstrated no statistical significance between the PbtO_2_ monitoring group and the ICP/CPP group. The discrepancy may stem from differences in the application and assessment methods of brain tissue oxygen monitoring and ICP/CPP between studies. We suggest that PbtO2 and ICP/CPP monitoring should be standardized, meanwhile, operators require better training to reduce the variations in results.

### Limitations

This study has some limitations. First, this study included two randomized controlled trials, while the remaining studies were retrospective cohort studies. Cohort studies have a greater risk of bias than randomized controlled trials. Second, since these retrospective studies were not randomized, the characteristics of patients included in the two evaluated groups may have varied. Additionally, different operators on the application of tissue oxygen monitoring is also diverse. Third, some studies selected a historical cohort as a control, which may have affected the comparability between studies. However, high-quality randomized controlled trials can address these limitations.

## Conclusions

In this systematic review and meta-analysis of 11 carefully selected studies, we compared PbtO_2_ monitoring with ICP/CPP monitoring in patients with TBI. Our analysis revealed that PbtO_2_ monitoring can decrease mortality and ICP but has no significant effect on and CPP. ICP/CPP monitoring appears to be associated with a short hospital length of stay, however, and we recommend this approach for patients with TBI as a potentially beneficial and effective intervention, as it allows for better monitoring and reduction of ICP contributing to a favorable neurological prognosis. The target population, intervention methods, implementation protocols, and high-quality randomized controlled trials are needed in large, well-designed studies and should be the focus of future research on the topic.

## Data Availability

No datasets were generated or analysed during the current study.
